# Pregnancy challenges and outcomes among female physicians

**DOI:** 10.15190/d.2024.11

**Published:** 2024-09-30

**Authors:** George Henning, Arjola Agolli, Susan Henning, Samantha Murphy

**Affiliations:** ^1^Pennsylvania State University, Penn State College of Medicine, Department of Family and Community Medicine, Hershey, PA; ^2^Penn State Health St Joseph Medical Center, Reading, PA; ^3^Pennsylvania State University, Penn State College of Medicine, Hershey, PA; ^4^Pennsylvania State University, Penn State College of Medicine, Department of Obstetrics and Gynecology, Hershey, PA

**Keywords:** Female physicians, infertility, pregnancy, pregnancy complications.

## Abstract

Female physicians constitute an increasing proportion of the total physician workforce. Lengthy training often causes delays in family planning. When they feel ready and plan to have children, they might face demanding work hours, limited options for parental leave and child support, and potential stigmatization by peers and superiors. The impact of these factors on female physicians’ fertility, pregnancy complications, professional growth, and perceptions of a career in medicine as a barrier to motherhood is not well-established. The goal of this study was to identify the main challenges and risk factors for pregnancy complications among U.S. female physicians. Age, stress, adverse working conditions, occupational hazards, and insomnia were some of the main factors that can affect female physicians' fertility. A higher rate of infertility and older age at delivery were observed among female physicians working in surgical specialties. Being a physician is often associated with higher rates of infertility and pregnancy complications than the general population. Although female physicians are increasing in number, they continue to encounter challenges in family planning and personal and professional life balance.  More research is needed to assess policy gaps, stigma, stereotypes, and risk factors, especially among different specialties. It becomes essential to develop effective strategies to adequately address these concerns and to offer equal and accessible reproductive care for female physicians.

## SUMMARY

1. Introduction
2. Physician gender disparity in parenthood
3. What are the challenges that female physicians encounter when planning a family?
4. Are the female physician’s pregnancies associated with higher risk for adverse pregnancy outcomes?
5. Pathophysiologic Mechanisms of Infertility and Pregnancy Adverse Outcomes among Female Physicians.
6. Discussion
7. Conclusions

## 1. Introduction

The percentage of female physicians within the total physician workforce has been rising. From 2007 to 2023, the percentage of physicians who identified as female increased from 28.3% to 37.6%^1^. Female physicians who plan to have children may face demanding work hours, limited options for parental leave and child support, and potential stigmatization by peers and superiors^2^. In addition, family responsibilities, poor human resource policies, and gender inequalities that slow professional growth are contributing factors that may lead to female physicians viewing a career in medicine as a barrier to motherhood^2,3^.

Female physicians have a long training path including medical school, residency training, and possibly fellowships. The average age at medical school graduation is 27.5 years, at completion of training (completion of medical school, residency, and/or fellowship) is 31.6 years, while the age at first pregnancy is 30.4 years^4^. However, increasing age negatively affects fertility. In female fetuses, oocyte count peaks at 20 weeks gestation. Oocyte numbers slowly decline from this point until 32 years old, at which point the decline in reserve becomes progressively faster^5^.

The incidence of infertility in the U.S. is estimated to be 9%–18% among the general population^6^ while 1 in 4 female physicians struggle with infertility^4^. Only 2% of female physicians had children before completing medical school. During residency training, family physicians have been reported to have similar rates of childbirth to nonphysicians, while specialists had lower rates of childbirth until they began independent practice^2^.

Pregnancies at older ages are associated with greater risks for complications during gestation and delivery^7^. Compared to 25- to 29-year-olds, females 35 years old and above had greater odds of preterm delivery, hypertension, severe preeclampsia, and superimposed preeclampsia. If 40 years old or greater at the time of delivery, risks increase for mild preeclampsia, poor fetal growth, and fetal distress^8^. Morbidity rates were 1.8 times higher in pregnant females 35 years old or above (127.9) compared with those at 20-24 years old (72.3)^9^. Between 2019 and 2021, the mortality rate in females aged 20 to 44 rose from 97.2 to 136.4 deaths per 100,000^9^.

The goal of our study is to identify the main factors for infertility and pregnancy complications among U.S. female physicians and areas with the greatest potential for improvement.

## 2. Physician gender disparity in parenthood

Among plastic surgeons, females were more likely than males to have no biological children (45.1% versus 23.1%). The findings included delayed childbirth due to demands of training (72.6% females versus 39.2% males) and were more likely to experience infertility (26.3% females versus 12.5% males). Among plastic surgeons who had no children, there were more females (20.1%) who responded they did not want to have children versus male plastic surgeons (1.8%)^10^. Among female oncologists, 1 in 3 reported experiencing infertility and 1 in 3 stated they experienced discrimination during pregnancy and/or for taking maternity leave^11^.

## 3. What are the challenges that female physicians encounter when planning a family?

Additionally, Canadian female otolaryngologists and neck surgeons report facing challenges in family planning, ability to conceive, and breastfeeding^12^.

There are several barriers related to fertility, family planning, and reproductive health among women physicians^13^. Female physicians appear to delay childbearing when compared to nonphysicians, and this phenomenon mostly occurs among specialists. Physicians seem to catch up to nonphysicians by starting reproduction at older ages and thus may be prone to increased risk of adverse reproductive outcomes^14^.

Female physicians face several barriers related to fertility, family planning, and reproductive health. Lai et al. collected survey responses from 4533 female physicians. Respondents were older at first pregnancy and had higher rates of infertility evaluation and treatment, as well as higher rates of miscarriage and preterm birth than the general population. Only 8% of those surveyed received education on the risks of delaying pregnancy during training. Those who were educated were significantly less likely to experience miscarriage or seek infertility evaluation or treatment. Physicians in nonsurgical specialties were younger than surgeons at the time of their first pregnancy, had fewer preterm births and fetal growth problems, and had more children^14^.

In one report, 25.5% of physicians encountered fertility issues, 72.9% of whom used fertility drugs, followed in prevalence by 54.2% who used fertility tracking. Among the main factors physicians reported for delaying pregnancy were demands of training (72.9%) and long work hours (61.5%)^13^.

Several studies have been conducted to shed light on pregnancy struggles and complications among females working in healthcare. The infertility rate among American female surgeons was more than twice the rate of the general population^15^. Often pregnancies were delayed due to training (65.0%), or required fertility treatment (24.9%), and experienced increased pregnancy complications when compared with the female partners of their nonchildbearing colleagues. Working more than 12 hours per week in surgery during pregnancy was associated with higher risks of pregnancy complications^15^. Surgeons experienced pregnancy loss (42.0%), stillbirth (3.8%), musculoskeletal disorders (36.9%), nonelective cesarean delivery (25.5%), and postpartum depression (11.1%)^15^.

More than half of all female surgeons delayed childbearing until they finished their training and were practicing independently^16^. Pregnancy during surgical training is associated with negative perceptions from peers^16,17^, pregnancy complications, and scheduling challenges^17^. However, in medical residency programs with female leadership, residents perceived a more supportive environment for having a pregnancy^18^. Out of 377 female physicians, 39.4% reported that their job prevented breastfeeding for the desired length of time or had significant workplace limitations to breastfeeding (52.2%).

Huguet et al., collected 398 responses from female otolaryngologists; their mean age at first pregnancy was 32.3 years, and 30.4% of them were diagnosed with infertility. When asked what they would do differently in retrospect, most women with infertility (65.0%) responded they would have attempted conception earlier; 41 (41.0%) would have used cryopreservation to extend fertility; and 14 (14.0%) would have gone into a different specialty^19^.

Similar findings were reported concerning female urologists. They also gave birth at older ages, had greater fertility issues, and used assisted reproductive technology. In addition, they reported a higher level of at least one complication during pregnancy when compared to U.S. females overall^20,21^. Female orthopedic surgeons had an increased risk of pregnancy complications, particularly preterm delivery, compared with the general U.S. population, especially among surgeons working more than sixty hours per week during pregnancy^22^.

For female physicians in procedural-based medical specialties, longer training and more intensive physical demands are required. Female physicians working in procedural fields perceived more challenges and were more likely to delay pregnancy when compared to female physicians non-proceduralists. However, rates of missed work due to pregnancy-related complications, reproductive assistance, and cesarean delivery were similar^23^. Those in procedural fields had greater rates of burnout and attrition from medicine, which the study attributed to difficulty balancing personal and professional demands^23^. A 2018 survey of the childbearing experiences during residency of anesthesiologists who completed a U.S. residency in 2000 or later found similar rates of pregnancy complications as the general U.S. population^24^. However, 65.7% of residents were discouraged from taking additional maternity leave and 51.6% of residents felt discouraged from being pregnant or having children, with rates not significantly changing over time^24^.

Female surgeons and physicians experience significantly greater infertility rates and pregnancy complications than other professional females. Delayed childbearing was observed in 65.0% of surgeons, 66.1% of non-surgeon physicians, and 57.5% of lawyers and those with other doctorates (*p*<0.001)^25^. Assisted reproductive technology was utilized by 24.9% of surgeons, 43.0% of non-surgeon physicians, and 21.7% of lawyers and those with other doctorates (*p*<0.001)^25^.

## 4. Are the female physician’s pregnancies associated with higher risk for adverse pregnancy outcomes?

Associations between physicians and pregnancy complications have been studied for over 30 years. In a 1990 study including three-year residencies (i.e. pediatrics, internal medicine, and family medicine), the rates of preterm delivery for women in postgraduate years 1, 2, and 3 were 8.4%, 3.2%, and 5.6%, respectively (p= 0.46)^26^. Corresponding rates of infants with small for gestational age at delivery were 4.2%, 5.7%, and 6.1% (p=0.53)^26^. Among residents, almost twice the rate of pre-eclampsia or eclampsia was observed, and this association did not change after adjustment for parity. Placental abruption was less likely to occur among residents (p =0.054)^26^. In these specialties, as training progresses physicians have fewer working hours and nights on-call which provides evidence against a strong effect of workload on pregnancy outcome, as no significant differences were found between years^26^. In the years since this study was performed, the structure of residency has changed, with work hours being restricted to 80 hours per week for residents.

Takeuchi et al. more recently studied the effect of long working hours and pregnancy complications among female physicians in Japan^27^. Out of the 939 subjects, 42% reported at least one complication during their first pregnancy. Threatened abortion (15%) and preterm birth (12%) were the most frequent complications, and (3.8%) of participants experienced both threatened abortion and preterm birth^27^. Family physicians, when compared to nonsurgical specialists (aOR, 1.12; 95% CI, 0.82-1.53) or surgical specialists (aOR, 1.43; 95% CI, 0.74-2.76), were not at increased risk of severe maternal morbidity^28^. Similar findings were observed for severe neonatal morbidity (nonsurgical specialists: aOR, 0.98; 95% CI, 0.80-1.19; surgical specialists: aOR, 1.08; 95% CI, 0.68-1.71)^28^. Findings showed that female physicians overall may be at a slightly higher risk of severe maternal morbidity and delayed childbearing when compared to nonphysicians^28^. Female plastic surgeons experienced seven times higher odds of having difficulty conceiving or carrying a pregnancy, twice the rate of miscarriage, and higher rates of obstetric complications and congenital malformations (47% vs. 20%, and 8% vs. 4%, respectively) when compared to the general U.S. population.^29^

Postpartum depression (PPD) among medical residents was another pregnancy complication reported. Female medical residents reported feeling PPD symptoms (42%) at more than four times the rate of PPD in the general population (11%). However, only 12% of them sought treatment or were diagnosed with PPD. Male residents did not report an increased rate of depressive symptoms; however, 19% of respondents believed their partner's symptoms were consistent with PPD^30^.

## 5. Pathophysiologic Mechanisms of Infertility and Pregnancy Adverse Outcomes among Female Physicians

**Age. **Increasing age is associated with decline of female fertility. Over time there are several factors that might affect fertility, such as decline in egg quality, reduced ovarian reserve, and increased frequency of health conditions^7^.

**Stress**. The stress system relates to all the major endocrine axes, including the reproductive axis. In males and females, the reproductive system can be inhibited by various components of the hypothalamic–pituitary–adrenal axis (HPA), at all levels. Corticotropin-releasing hormone (CRH) suppresses the gonadotropin-releasing hormone (GnRH) neurons directly and indirectly via enhancing β-endorphin secretion by the arcuate pro-opiomelanocortin (POMC) neurons. Corticotropin-releasing hormone receptor 1 (CRH-R1) partially mediates the effects of restraint acute stress on the reproductive axis, whilst antalarmin (a selective CRH-R1 antagonist) can stop these effects. In addition, glucocorticoids serve as inhibitors of GnRH neurons, pituitary gonadotrophs, and the gonads, and cause resistance to sex hormones on target organs and tissues. On the hypothalamic level, there is observed inhibition of the pulsatile GnRH secretion and concomitantly steroidogenesis is directly inhibited at the level of ovaries and testes. Specific pro-inflammatory circulating cytokines can also remarkably suppress the reproductive function at multiple levels, providing a link between reproductive dysfunction and inflammatory stress^31^.

The inhibitory effect at the hypothalamic level can cause functional hypothalamic amenorrhea especially in situations of eating disorders, chronic excessive exercise, and prolonged or chronic stress^32^. In women exposed to high psychosocial stress, the ovarian CRH is also one of the potential mechanisms implicated in the premature ovarian failure^33^. CRH can also affect embryo implantation and pregnancy maintenance by killing activated T-cells and regulating the expression of carcinoembryonic antigen-related cell adhesion molecule-1 (CEACAM1)^34^.

Prenatal maternal stress can also reduce the activity of placental 11β-hydroxysteroid dehydrogenase type 2 (11β-HSD 2), thus allowing high circulating levels of maternal glucocorticoids to enter the fetal circulation. As a result, it can cause early and long-term developmental effects resulting in part from altered maternal and/or fetal glucocorticoid exposure^35^. Maternal stress, depression, and anxiety in pregnancy can affect “fetal programming” in a child. Animal models used for research indicate that maternal distress negatively influences long-term learning, motor development, and behavior in offspring^36^.

Stress has the potential to impair fertility during critical periods involving regular menstruation, successful ovulation, implantation, and placental growth and development^37^. Among female physicians 26-37 weeks pregnant, urinary catecholamines increased by 58% (p <0.03) during work periods compared with non-work periods^38^. An increase of 64% (p<0.025) occurred in the working non-physician control group of similar gestational age^38^. Catecholamine levels can decrease uterine blood flow and tend to reach higher levels during physical stress, such as standing, and mental stress, such as difficult problem-solving.

**Occupational hazards and working conditions**. Adverse pregnancy outcomes among healthcare workers have been linked to poor working conditions including long working hours, prolonged standing, lifting heavy objects, and psychological stress. Also, poor ergonomic practices (factors likely to affect interactions between an individual and their working environment) often increase existing discomfort, which can cause increased rates of work absenteeism, sick leave, and a lack of motivation among pregnant healthcare workers^39^.

Overtime work among female nurses was associated with an increased risk of threatened abortion, while handling disinfectant was associated with an increased risk of threatened abortion and spontaneous abortion. In addition, nurses handling anti-cancer drugs were found to have an almost twofold increased risk of premature birth^40^.

Several occupational hazards, such as radiation exposure (at >100 millirems), infectious bioagents, heavy lifting, and prolonged working hours (>12 hours per shift), can negatively impact a developing fetus^41^. Sources of X-rays and gamma rays can cause birth defects, low birth weights, infertility, miscarriage, and other developmental disorders^42^. In addition, sharps injury, anesthetic gases, radiation, surgical smoke, working conditions, and intraoperative use of toxic agents can affect physicians working in the operating room. Studies have shown that surgeons have higher rates of spontaneous abortion, preterm delivery, growth restriction, congenital abnormalities, infertility, and pregnancy complications when compared with the general population^43^.

Pregnant staff working in the orthopedic operating room may be at risk of occupational exposure to several occupational hazards thought to be a threat to fetal development, such as anesthetic gases, methylmethacrylate, blood-borne pathogens, physical stress, and radiation^44^. However, very few studies have been conducted on the physician population regarding exposure to anesthetic gases^45^. Additionally, it is not clear whether modern anesthetic gas scavenging systems are sufficient to prevent exposure to operating room personnel^46^, and particularly, anesthesiologists. When comparing pregnancy complications and losses between attending anesthesiologists and obstetricians, 65.1% of anesthesiologists and 65.5% of obstetricians reported at least one pregnancy complication, and 36% and 24% reported at least one pregnancy loss, respectively^47^. There was no significant difference between groups in terms of overall complication rate or types of pregnancy complication, although rates of complications and losses are higher than those of both the general population and other specialties in previous reports^47^. Pediatric anesthesia more often requires induction with inhaled gases as compared to adult anesthesia, exposing pediatric anesthesiologists to higher levels of anesthetic gases. Gauger et al compared pregnancy complications among pediatric anesthesiologists to those among adult anesthesiologists and found a significantly higher prevalence of spontaneous abortion in pediatric anesthesiologists. There was no significant difference observed in any other pregnancy complications between the two groups^48^.

**Insomnia. **Insomnia may co-occur with, or result in, HPA activation, thereby leading to infertility. Patients with chronic insomnia were found to have elevated stress hormone responses when compared to people who have good sleep. Sleep dysregulation may alter successful conception through the suppression or augmentation of reproductive hormones and may affect conception via compromised immunity (sleep loss is associated with increased tumor necrosis factor (TNF) and interleukin-6 (IL-6), as well as elevated C-reactive protein (CRP) is found in young women with poor sleep). There is some evidence that links circadian influence with shift work and infertility^37^.

**Other factors**. Some other common factors that affect fertility in the general population are smoking, excessive alcohol use, overweight/obesity or underweight, extreme weight gain or loss, and excessive physical or emotional stress^7^.

## 6. Discussion

Evidence-based research shows that female physicians may be more likely than their male colleagues to adhere to clinical guidelines, provide more psychosocial counseling to their patients, and provide preventive care more often, including cancer screening services.They tend to use more patient-centered communication and are likely to spend more time with their patients than male physicians. They more often adopt a partnership-building style with patients^49^. Training necessary to practice independently is long. The average age at completion of training is 31.6 years, and physicians tend to have their first pregnancy around 30.4 years old^4^. Older age affects female fertility with a progressive decline in egg quality, and reduced ovarian reserve, as well as increased incidence of other health conditions that also can affect fertility^7^. The causes of infertility and the pregnancy outcomes among female physicians remain understudied and unaddressed. System-level interventions should be considered to support female physicians who wish to have children at all career stages^2^.

The share of physicians in the U.S. that would recommend medicine as a career to young people from 2014 to 2021 declined from 59% to 46%^50^. Inadequate support in the workplace environment can lead to an unbalanced equilibrium between professional and personal life and also affect family responsibilities. In addition, stereotyping and discrimination increase stress levels, lower work productivity, and lower life satisfaction. Low life satisfaction can negatively affect both workers’ personal lives, as well as their professional productivity and work quality. It is essential to consider reducing long working hours, offering flexible timing and part-time work options that might contribute to increasing the motivation of females working in the healthcare workplace^3^.

Between 2019 and 2022 there was observed an 8.3-minute increase per year in the already existing gap between the time men and women physicians spend on the Electronic Health Record (EHR) for every eight hours of scheduled clinic time. Male physicians spent 5.3 hours interacting with the EHR while women physicians spent an average of 6.4 hours in the EHR for every eight hours of patient scheduled time^51^. This disparity in work outside of work is concerning given its association with physician burnout. A root cause analysis must be done to explore the causes behind these differences and develop solutions to address them, considering the higher rates of burnout among female physicians.

Among female physicians who had young children and reported support from their colleagues, spouses, or significant others at work and at home, the odds of burnout decreased by 40%^52^. Having a pregnancy during medical residency can be challenging. Pregnant residents are more likely to be placed on bed rest for high-risk pregnancies than the general population^26^.A survey study during the years 2012-2013, collected data from a sample of 600 female physicians. A substantial proportion of them have faced infertility or have regrets about family planning and career decision-making^4^. Having a medical career and combining it with motherhood continues to be challenging and deserves further investigation and targeted support.

The most commonly reported concerns during pregnancy among health professionals are neck pain, nausea, vomiting, back pain, and pelvic girdle pain. Working night shifts increases the risk for having pregnancy complications such as miscarriage, threatened abortion, premature labor, and hypertensive disorders of pregnancy (HDP), and the risk increases with the number and length of shifts^53^.

For females working in healthcare, inconsistent maternity protection laws, lack of paid parental leave, financial losses, and career opportunities during pregnancy, can pose additional challenges. In addition, interpersonal relationships and difficulties in adapting to work during pregnancy and postnatally often add to the already substantial time and effort involved in breastfeeding. Pregnancy and gender discrimination in the workplace should be recognized. It is important to provide specific maternity protection rules in occupational health and safety legislation at the national level^53^. When having a family, female physicians are more likely than male physicians to work part-time or discontinue working completely^54^. Inconsistent and unclear maternity protection policies, such as paid parental leave, can financially penalize families, reduce career opportunities and productivity, and make breastfeeding difficult^55^. There is a lack of access to adequate insurance coverage for this higher risk group^56^. Evidence-based research shows that up to 25% of female physicians are seeking assisted reproductive technology^56^. Currently, 1 cycle of assisted reproductive technology, or egg retrieval, is estimated to cost approximately $19,000, with many patients needing multiple cycles^56^. Only a few institutions offer coverage for those seeking oocyte or embryo cryopreservation for elective fertility preservation^56^. Cryopreservation is another emerging request being considered by female physicians. Females (46.0%) are more likely than males (23.7%) to state that employer-sponsored covered planned oocyte cryopreservation would influence their residency ranking.^57^. Less is known to what extent employer-sponsored covered cryopreservation would determine medical career decision-making^57^.

These findings suggest that different strategies may be needed to improve the work lives of female physicians.

## 7. Conclusions

Studies indicate that family planning at an older age, long working hours, physical and psychological stress, occupational hazards and lack of full support from employers and colleagues are some of the main factors related to increased risk for adverse events during pregnancy among female physicians. Childbearing age coincides with peak medical training, causing a career in medicine to be viewed as barrier to motherhood. Infertility treatments are often required and only few institutions offer coverage.

More research is required to better understand all the factors related to infertility and pregnancy complications among female physicians. With increasing numbers of female physicians in the healthcare industry, it has become essential to develop effective strategies to adequately address these concerns and to offer equal and accessible reproductive care for female physicians. This should lead to consistent, research-informed policies pertaining to fertility, pregnancy and parenting for physicians and healthcare professionals.

## References

[1] U.S. Physician Workforce Data Dashboard. Association of American Medical Colleges.

[2] Cusimano Maria C, Baxter Nancy N, Sutradhar Rinku, McArthur Eric, Ray Joel G, Garg Amit X, Vigod Simone, Simpson Andrea N (2021). Delay of Pregnancy Among Physicians vs Nonphysicians.. JAMA internal medicine.

[3] ALobaid Abdullah Mohammed, Gosling Cameron McR, Khasawneh Eihab, McKenna Lisa, Williams Brett (2020). Challenges Faced by Female Healthcare Professionals in the Workforce: A Scoping Review.. Journal of multidisciplinary healthcare.

[4] Stentz Natalie Clark, Griffith Kent A, Perkins Elena, Jones Rochelle DeCastro, Jagsi Reshma (2016). Fertility and Childbearing Among American Female Physicians.. Journal of women's health (2002).

[5] (2014). Female age-related fertility decline. Committee Opinion No. 589.. Fertility and sterility.

[6] Chandra Anjani, Martinez Gladys M., Mosher William D., Abma Joyce C., Jones Jo (2005). Fertility, family planning, and reproductive health of U.S. women: Data from the 2002 National Survey of Family Growth. PsycEXTRA Dataset.

[7] (2023). Division of Reproductive Health, National Center for Chronic Disease Prevention and Health Promotion. Centers for Disease Control and Prevention. Infertility.

[8] Cavazos-Rehg Patricia A, Krauss Melissa J, Spitznagel Edward L, Bommarito Kerry, Madden Tessa, Olsen Margaret A, Subramaniam Harini, Peipert Jeffrey F, Bierut Laura Jean (2015). Maternal age and risk of labor and delivery complications.. Maternal and child health journal.

[9] (2023). United Health Foundation. 2023 Health of Women and Children Report. America’s Health Rankings.

[10] Furnas Heather J, Li Alexander Y, Garza Rebecca M, Johnson Debra J, Bajaj Anureet K, Kalliainen Loree K, Weston Jane S, Song David H, Chung Kevin C, Rohrich Rod J (2019). An Analysis of Differences in the Number of Children for Female and Male Plastic Surgeons.. Plastic and reconstructive surgery.

[11] Lee Anna, Kuczmarska-Haas Aleksandra, Dalwadi Shraddha M, Gillespie Erin F, Ludwig Michelle S, Holliday Emma B, Chino Fumiko (2022). Family Planning, Fertility, and Career Decisions Among Female Oncologists.. JAMA network open.

[12] Naismith Kendra, Ioanidis Khrystyna, Dzioba Agnieszka, MacNeil S Danielle, Paradis Josee, Nayan Smriti, Strychowsky Julie E, Graham M Elise (2023). Canadian women in otolaryngology: head and neck surgery part 2-challenges in family planning, fertility, and lactation.. Journal of otolaryngology - head & neck surgery = Le Journal d'oto-rhino-laryngologie et de chirurgie cervico-faciale.

[13] Armijo Priscila Rodrigues, Flores Laura, Huynh Linda, Strong Sheritta, Mukkamala Shivani, Shillcutt Sasha (2021). Fertility and Reproductive Health in Women Physicians.. Journal of women's health (2002).

[14] Lai Krista, Garvey Erin M, Velazco Cristine S, Gill Manrit, Weidler Erica M, van Leeuwen Kathleen, Kim Eugene S, Rangel Erika L, Grimsby Gwen M (2023). High Infertility Rates and Pregnancy Complications in Female Physicians Indicate a Need for Culture Change.. Annals of surgery.

[15] Rangel Erika L, Castillo-Angeles Manuel, Easter Sarah Rae, Atkinson Rachel B, Gosain Ankush, Hu Yue-Yung, Cooper Zara, Dey Tanujit, Kim Eugene (2021). Incidence of Infertility and Pregnancy Complications in US Female Surgeons.. JAMA surgery.

[16] Turner Patricia L, Lumpkins Kimberly, Gabre Joel, Lin Maggie J, Liu Xinggang, Terrin Michael (2012). Pregnancy among women surgeons: trends over time.. Archives of surgery (Chicago, Ill. : 1960).

[17] Mavedatnia Dorsa, Ardestani Shakiba, Zahabi Sarah, Neocleous Penelope, Madou Edward, Dzioba Agnieszka, Strychowsky Julie E, Graham M Elise (2023). The Experiences of Motherhood in Female Surgeons: A Scoping Review.. Annals of surgery.

[18] Mundschenk Minh-Bao, Krauss Emily M, Poppler Louis H, Hasak Jessica M, Klingensmith Mary E, Mackinnon Susan E, Tenenbaum Marissa M (2016). Resident perceptions on pregnancy during training: 2008 to 2015.. American journal of surgery.

[19] Huguet Makenzie, Beliveau Angela, Taylor Sandra L, Aizenberg Debbie A (2022). Pregnancy and Fertility Trends Among Female Otolaryngologists.. Otolaryngology--head and neck surgery : official journal of American Academy of Otolaryngology-Head and Neck Surgery.

[20] Scott Victoria C S, Lerner Lori B, Eilber Karyn S, Anger Jennifer T, Ackerman A Lenore (2020). Re-evaluation of birth trends and pregnancy complications among female urologists: Have we made any progress?. Neurourology and urodynamics.

[21] Lerner Lori B, Stolzmann Kelly L, Gulla Vanessa D (2009). Birth trends and pregnancy complications among women urologists.. Journal of the American College of Surgeons.

[22] Hamilton Abigail R, Tyson Mark D, Braga Julie A, Lerner Lori B (2012). Childbearing and pregnancy characteristics of female orthopaedic surgeons.. The Journal of bone and joint surgery. American volume.

[23] Scully Rebecca E, Stagg Amy R, Melnitchouk Nelya, Davids Jennifer S (2017). Pregnancy outcomes in female physicians in procedural versus non-procedural specialties.. American journal of surgery.

[24] Kraus Molly B, Thomson Holly M, Dexter Franklin, Patel Perene V, Dodd Sarah E, Girardo Marlene E, Hertzberg Linda B, Pearson Amy C S (2021). Pregnancy and Motherhood for Trainees in Anesthesiology: A Survey of the American Society of Anesthesiologists.. The journal of education in perioperative medicine : JEPM.

[25] Yau Alice, Lentskevich Marina A, Ahmed Kaleem S, Rangel Erika L, Gosain Arun K (2024). Female Surgeons and Physicians Experience Greater Infertility Rates and Pregnancy Complications Than Other Professional Women.. The American surgeon.

[26] Klebanoff M A, Shiono P H, Rhoads G G (1990). Outcomes of pregnancy in a national sample of resident physicians.. The New England journal of medicine.

[27] Takeuchi Masumi, Rahman Mahbubur, Ishiguro Aya, Nomura Kyoko (2014). Long working hours and pregnancy complications: women physicians survey in Japan.. BMC pregnancy and childbirth.

[28] Cusimano Maria C, Baxter Nancy N, Sutradhar Rinku, McArthur Eric, Ray Joel G, Garg Amit X, Vigod Simone, Simpson Andrea N (2022). Evaluation of Adverse Pregnancy Outcomes in Physicians Compared With Nonphysicians.. JAMA network open.

[29] Hemal Kshipra, Chen Wendy, Bourne Debra A (2023). Fertility and Childbearing Outcomes of Practicing Female Plastic Surgeons.. Plastic and reconstructive surgery.

[30] Bye Emma, Leval Rebecca, Sayles Harlan, Doyle Marley, Mathes Melissa, Cudzilo-Kelsey Laura (2022). Parental postpartum depression among medical residents.. Archives of women's mental health.

[31] Tsigos Constantine, Kyrou Ioannis, Kassi Eva, Chrousos George Stress: Endocrine Physiology and Pathophysiology.

[32] Valsamakis Georgios, Chrousos George, Mastorakos George (2019). Stress, female reproduction and pregnancy.. Psychoneuroendocrinology.

[33] Bromberger J T, Matthews K A, Kuller L H, Wing R R, Meilahn E N, Plantinga P (1997). Prospective study of the determinants of age at menopause.. American journal of epidemiology.

[34] Kalantaridou Sophia N., Makrigiannakis Antonis, Zoumakis Emmanouil, Chrousos George P. The Role of Corticotropin-Releasing Hormone (CRH) on Implantation and Immunotolerance of the Fetus. Medical Intelligence Unit.

[35] Mairesse Jérôme, Lesage Jean, Breton Christophe, Bréant Bernadette, Hahn Tom, Darnaudéry Muriel, Dickson Suzanne L, Seckl Jonathan, Blondeau Bertrand, Vieau Didier, Maccari Stefania, Viltart Odile (2007). Maternal stress alters endocrine function of the feto-placental unit in rats.. American journal of physiology. Endocrinology and metabolism.

[36] Dunkel Schetter Christine, Tanner Lynlee (2012). Anxiety, depression and stress in pregnancy: implications for mothers, children, research, and practice.. Current opinion in psychiatry.

[37] Kloss Jacqueline D, Perlis Michael L, Zamzow Jessica A, Culnan Elizabeth J, Gracia Clarisa R (2015). Sleep, sleep disturbance, and fertility in women.. Sleep medicine reviews.

[38] Katz V L, Jenkins T, Haley L, Bowes W A (1991). Catecholamine levels in pregnant physicians and nurses: a pilot study of stress and pregnancy.. Obstetrics and gynecology.

[39] Francis Frincy, Johnsunderraj Sheeba E, Divya K Y, Raghavan Divya, Al-Furgani Atiya, Bera Lily P, Abraham Aniamma (2021). Ergonomic Stressors Among Pregnant Healthcare Workers: Impact on pregnancy outcomes and recommended safety practices.. Sultan Qaboos University medical journal.

[40] Jiang Zhaoqiang, Chen Junfei, Feng Lingfang, Jin Mingying, Liu Shuang, Wang Lina, Wang Jing, Yu Changyan, Zhou Jianhong, Ye Yan, Mei Liangying, Yu Wenlan, Zhang Xing, Lou Jianlin (2023). Associations between maternal occupational exposures and pregnancy outcomes among Chinese nurses: a nationwide study.. Reproductive health.

[41] Lawson Christina C, Whelan Elizabeth A, Hibert Eileen N, Grajewski Barbara, Spiegelman Donna, Rich-Edwards Janet W (2009). Occupational factors and risk of preterm birth in nurses.. American journal of obstetrics and gynecology.

[42] Shaw Palma, Duncan Audra, Vouyouka Ageliki, Ozsvath Kathleen (2011). Radiation exposure and pregnancy.. Journal of vascular surgery.

[43] Anderson Matilda, Goldman Rose H (2020). Occupational Reproductive Hazards for Female Surgeons in the Operating Room: A Review.. JAMA surgery.

[44] Downes Jessica, Rauk Philip N, Vanheest Ann E (2014). Occupational hazards for pregnant or lactating women in the orthopaedic operating room.. The Journal of the American Academy of Orthopaedic Surgeons.

[45] Delva Fleur, Carcasset Pierre, Mouton Pauline, Auguste-Virginie Rivana, Lairez Fanny, Sentilhes Loïc, Brochard Patrick, Joseph Jean-Philippe (2022). Greater Risk of Pregnancy Complications for Female Surgeons: A Cross-Sectional Electronic Survey.. International journal of environmental research and public health.

[46] García-Álvarez José Manuel, Escribano-Sánchez Guillermo, Osuna Eduardo, Molina-Rodríguez Alonso, Díaz-Agea José Luis, García-Sánchez Alfonso (2023). Occupational Exposure to Inhalational Anesthetics and Teratogenic Effects: A Systematic Review.. Healthcare (Basel, Switzerland).

[47] Barnett Natalie R, George Renuka M, Hatter Katherine H, Janosy Norah R, Vizzini Samantha J, Singh Shubhangi, Lee Rebecca E, Wolf Bethany J, Cabrera Camila, Duhachek-Stapelman Amy L, Katz Daniel (2024). Pregnancy complications and loss: an observational survey comparing anesthesiologists and obstetrician-gynecologists.. The journal of maternal-fetal & neonatal medicine : the official journal of the European Association of Perinatal Medicine, the Federation of Asia and Oceania Perinatal Societies, the International Society of Perinatal Obstetricians.

[48] Gauger Virginia T, Voepel-Lewis Terri, Rubin Philip, Kostrzewa Amy, Tait Alan R (2003). A survey of obstetric complications and pregnancy outcomes in paediatric and nonpaediatric anaesthesiologists.. Paediatric anaesthesia.

[49] Joseph Madeline M, Ahasic Amy M, Clark Jesse, Templeton Kim (2021). State of Women in Medicine: History, Challenges, and the Benefits of a Diverse Workforce.. Pediatrics.

[50] Yang Jenny (2024). Share of physicians in the U.S. that would recommend medicine as a career to young people from 2014 to 2023. Statista.

[51] Henry Tanya Albert (2023). EHR gender gap widens as women physicians spend more time on task. American Medical Association.

[52] McMurray J E, Linzer M, Konrad T R, Douglas J, Shugerman R, Nelson K (2000). The work lives of women physicians results from the physician work life study. The SGIM Career Satisfaction Study Group.. Journal of general internal medicine.

[53] Vasconcelos Soraya Wingester, Guedes Joana C, Dias Elizabeth Costa, Matias Alexandra (2023). Pregnancy and working conditions in the hospital sector: a scoping review.. Revista brasileira de medicina do trabalho : publicacao oficial da Associacao Nacional de Medicina do Trabalho-ANAMT.

[54] Frank Elena, Zhao Zhuo, Sen Srijan, Guille Constance (2019). Gender Disparities in Work and Parental Status Among Early Career Physicians.. JAMA network open.

[55] Gottenborg Emily, Maw Anna, Ngov Li-Kheng, Burden Marisha, Ponomaryova Anastasiya, Jones Christine D (2018). You Can't Have It All: The Experience of Academic Hospitalists During Pregnancy, Parental Leave, and Return to Work.. Journal of hospital medicine.

[56] Veade Ashley, Martin Caitlin, Dombrowski Michael, Omurtag Kennan (2023). Female physician infertility: the lack of adequate insurance coverage.. American journal of obstetrics and gynecology.

[57] Murphy Hana G, Compton Sarah D, Moravek Molly B, Rosen Monica W (2024). Impact of employer-covered planned oocyte cryopreservation on decision-making for medical training.. Journal of assisted reproduction and genetics.

